# Surface Nano-Patterning for the Bottom-Up Growth of III-V Semiconductor Nanowire Ordered Arrays

**DOI:** 10.3390/nano11082079

**Published:** 2021-08-16

**Authors:** Valeria Demontis, Valentina Zannier, Lucia Sorba, Francesco Rossella

**Affiliations:** 1 NEST, Scuola Normale Superiore and Istituto Nanoscienze CNR, Piazza S. Silvestro 12, 56127 Pisa, Italy; valentina.zannier@nano.cnr.it (V.Z.); lucia.sorba@nano.cnr.it (L.S.); 2 Dipartimento di Scienze Fisiche, Informatiche e Matematiche, Università di Modena e Reggio Emilia, Via Campi 213/A, 41125 Modena, Italy

**Keywords:** semiconductor nanowire ordered arrays, substrate patterning, nanowire metasurfaces

## Abstract

Ordered arrays of vertically aligned semiconductor nanowires are regarded as promising candidates for the realization of all-dielectric metamaterials, artificial electromagnetic materials, whose properties can be engineered to enable new functions and enhanced device performances with respect to naturally existing materials. In this review we account for the recent progresses in substrate nanopatterning methods, strategies and approaches that overall constitute the preliminary step towards the bottom-up growth of arrays of vertically aligned semiconductor nanowires with a controlled location, size and morphology of each nanowire. While we focus specifically on III-V semiconductor nanowires, several concepts, mechanisms and conclusions reported in the manuscript can be invoked and are valid also for different nanowire materials.

## 1. Introduction

Semiconductor nanowires have emerged as a powerful class of materials with great potential for applications in many fields of technology, due to their outstanding electronic and optical properties and a relatively easy, controllable and scalable bottom-up growth process [1,2]. Their employment at large scale sets challenging requirements in terms of position, size and shape control of the individual nano-objects in nanowire ensembles [3,4]. This level of control represents a necessary step towards the realization of reliable and reproducible devices exploiting a very large number of nanowires for their operation, and for the integration of nanowire-based systems and devices with other technological platforms, such as silicon electronics and photonics [5,6]. Ordered arrays of vertically aligned semiconductor nanowires (NWs) have gained significant attention over the last two decades, as they have emerged as promising platforms in several fields of research including optics [7,8,9], electronics [10,11,12], energy [13,14,15,16], quantum computing [17,18], integrated photonics [19,20], sensing [21,22,23], and life sciences [24,25].

By exploiting the advances in nanofabrication and growth techniques, nanowire assemblies can nowadays be finely designed in order to fully tune and adjust their physical properties [26]. In particular, nanowire arrays can be engineered to realize nanowire metamaterials and metasurfaces, artificially prepared electromagnetic materials made of resonant subwavelength structures, showing effective medium properties that do not exist in nature. Photonic metamaterials made of assemblies of metal or semiconductor NWs have been experimentally and theoretically investigated [27,28]: nanowire metamaterials with optical negative refraction [29], optical cloaks [30], electrically tunable metamaterials [31], and near zero reflectance materials [32] have been reported.

In general, nanowire assemblies may have several properties which exceed those of their thin film counterparts [1,2]. These structures benefit from the large aspect ratios (length/diameter) of the constitutive NWs, which generate a strong optical anisotropy in the composite [33], which can be properly tuned in order to effectively control the light propagation in the medium. Nanowires, moreover, support resonant optical modes which can be tailored and enhanced in order to modulate the reflectance, absorbance and transmittance properties of the arrays [9,13,15,34,35]. Nanowire arrays also provide a large surface-to-volume area, beneficial in all the applications based on surface interactions mechanisms, such as sensing [36], photodetection [37], and batteries electrodes [38], to name a few.

As shown in the following sections, nanowires are available in a large variety of materials and they can incorporate heterostructures made of different components, even combining materials that are challenging to be combined in their thin film counterparts.

In this review we shine light on the recent progresses in the bottom-up realization of ordered arrays of vertically arranged semiconductor nanowires and nanowire metasurfaces by systematically reviewing the role of substrate pre-patterning, the current opportunities and the experimental limitations. Indeed, the realization of highly ordered nanowire arrays requires a preliminary step of substrate lithographic patterning, aimed at defining the specific nanowire locations on the substrate. Notably, the geometry and quality of the pattern employed to assist the nanowire growth dramatically affects the characteristics of the grown nanowire array. This study pinpoints and fixes the key concepts and phenomena guiding the subtle interdependence between surface pre-patterning and nanowire array growth. Such concepts and phenomena are typically common to several nanowire materials and growth approaches. In our study we devote particular attention to nanowire systems made of III-V semiconductor compounds; however, several mechanisms discussed for III-V’s can be invoked with minor differences for other semiconductors and semiconductor compounds.

It is also worth noting that, among the III-V semiconductor compounds, we focus our attention on III-arsenides (III-As) and III-phosphides (III-P), rather than on III-nitrides. The latter represent indeed a class of semiconductors often regarded as a unique material inside the III-Vs family. In fact, on the one hand they display an hexagonal wurtzite structure with strong polarization effects that are typically not so relevant for the other III-Vs. On the other hand, their synthesis needs very high growth temperatures and growth rates, and their properties very strongly depend on a plethora of external conditions including doping, substrate material, density of structural defects, and strain fields. In addition, while III-nitride NW arrays raise interest mainly for their optoelectronic and electronic applications including visible LEDs, sensors, FETs, and piezoelectric harvesters, III-As/P NW assemblies are deserving special attention for the engineering of optical metamaterials and metasurfaces, which is currently driving interest in applications, motivating the present review. However, the bottom-up strategies to obtain nitride NW arrays are conceptually not dissimilar to those exploited for their III-V counterpart, which will be described in Section 3, and, at a time, surface patterning and the role of the pattern characteristics in the final NW shape and aspect ratio as described in Section 4 are general and valid for different III-V compound semiconductors.

## 2. Semiconductor Nanowires and Nanowire Arrays

Since their first discovery in the late 1960s [39], semiconductor NWs have been studied extensively and research in this field has now reached a high level of maturity. NWs ordered arrays can be fabricated by several methods and can be categorized into two different approaches: top-down and bottom-up. Top-down fabrication is a subtractive technique which consists of carving the NWs from a bulk material, by means of anisotropic etching, according to previously defined lithographic patterns on the bulk sample’s surface. Top-down processing is of course mature and scalable and quite versatile, and it allows NW patterning with relatively high reproducibility and sharp size distribution [40]. However, in order to reach full control over the structural and morphological features of the single nano-objects, including defect distribution and quality of the nanowire surface, thus achieving a very high quality of the NW array as a whole, top-down approaches do require an extremely fine control over the etching processes, which represents a very challenging task, if not a formidable problem. In addition, and more drastically, top-down methods pose serious restraints regarding the possibilities to explore different structures and semiconductor materials and a combination of the two, preventing engineering of complex NW heterostructures and ultimately limiting dramatically the properties and phenomena at the level of the single nano-objects and of the entire array. For instance, the growth of NWs made of a semiconductor material with a significant lattice mismatch with respect to the semiconductor substrate is an extremely challenging task [5].

Remarkably, all the issues mentioned above can actually be fixed by resorting to the use of bottom-up growth methods, which represent, in fact, the most widely exploited techniques for the realization of ordered arrays of semiconductor NWs: for these reasons, in this review we will focus on the arsenal of surface nano-patterning methods that are required for the bottom-up growth of NW arrays.

A fundamental boost in nanowire research originated from the pioneering works of Lieber, Yang and Samuelson in the ‘90s, who were the first to demonstrate the possibility to realize, by using bottom-up self-assembling growth techniques, very high quality nanowires structures [41,42,43,44], thus sparking the interest of the scientific community in this field. In particular, they demonstrated the versatility of the bottom-up growth in enabling the realization, during growth, of very high quality nanowire crystals and heterostructures, made of combinations of different materials, resulting in very abrupt and atomically sharp interfaces. This opportunity is permitted by the strain relaxation along the nanowire sidewalls occurring in bottom-up growth, enabling the coupling of materials with a high lattice constant mismatch, such as, for example, III-V materials on silicon substrates [1,45,46]. Moreover, self assembling generally enables the realization of less defected surfaces and a better control of the resulting facets [1] with respect to top-down lithography approaches. These advantages made bottom-up self-assembling the most eligible approach for nanowire growth.

Research in the nanowire field evolved very quickly in the last decades, as the growth process versatility opened the way to the realization and investigation of a large variety of nanowire devices, with a plethora of unprecedented functionalities. Today, both individual nanowires and nanowire assemblies are considered as established test-bed platforms for the investigation of outstanding device functional properties and for the engineering of materials at the nanoscale. Among all the successful applications reported in the literature, here we name just a few examples in order to provide an idea of the potential of these systems both in research and technology. Single electron transistors made of axial InAs/InP nanowire heterostructures (quantum dots) [47,48], axial and radial NW heterostructures applied to solar cells [16] and other core-shell devices [49,50], light emitting diodes [51], lasers [52], photodetectors [53,54], thermoelectric devices [55,56], sensors [57], spin based quantum systems [58,59,60], topological qubits based on Majorana physics [61,62], and many others have been reported. Moreover, homogeneous nanowires and heterostructured nanowires are widely employed as test-bed platforms for investigating basic physics phenomena, including properties of materials [63,64,65], ion gating mechanisms [66,67,68,69], advanced quantum concepts [45], hybrid semiconductor-superconductors systems [70,71], etc.

Many of the above cited papers report results obtained by using nanowires made of III-V semiconductor compounds. These systems are indeed largely investigated due to their peculiar properties such as the low electron effective mass and high electron mobility, which makes them suitable for high speed electronic devices, their direct energy band gap, which makes them efficient in light emission and other optic and photonic applications [72,73] and the strong spin orbit coupling, which makes them promising candidates for spintronic applications and topological quantum computation [74]. Their integration into silicon platforms is considered, at present, to be one of the most interesting challenges for the next decade of NW research [3,75], as this would allow a combination of the advantages of III-V materials with the well-developed silicon technologies.

In recent years, new avenues for exploration in the nanowire field have been enabled by the advances in growth and nanofabrication techniques, which has allowed the design of engineered nanowire patterns with a fine control over the size, shape, material composition, location, and orientation of each individual nanowire.

Apart from the interest related to the exploration of new physical phenomena, such as advanced mechanisms of light propagation control at the nanoscale, the deterministic growth at selected locations on the substrate of nanowires represents the fundamental challenge to be addressed from the perspective of the full exploitation of nanowire potentialities in large scale applications, possibly with their integration with more mature (e.g., silicon-based) technological platforms [3,4].

**Figure 1:**
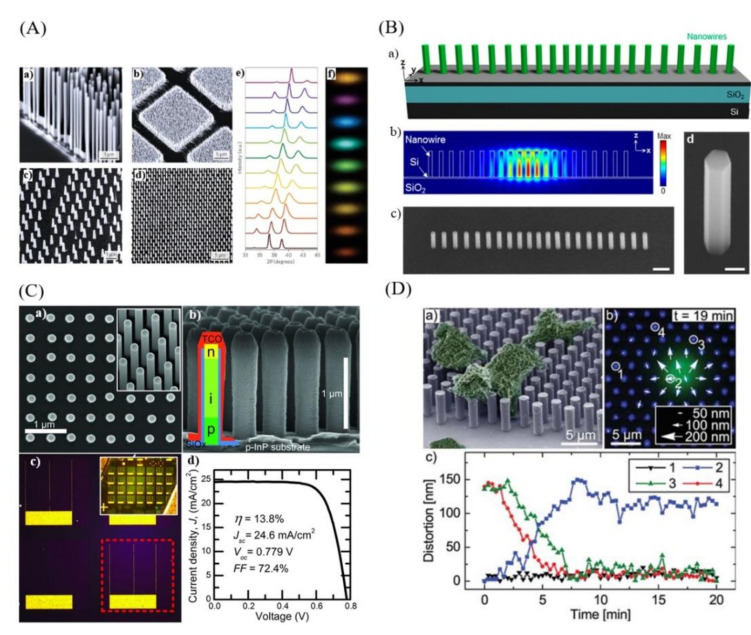
(**A**) Scanning-electron microscope images of different vertically aligned semiconductor nanowire arrays (ZnO NWs (a), GaN NWs (b), InP NW (c), InAs/InP heterostructured NWs (d)), (e) X-ray diffraction for InGaN nanowires arrays with different In concentrations, (f) CCD camera image of the visible photoluminescence emission of In_x_Ga_1−x_N nanowire (x = 0–0.6) (reprinted with permission of Refs. [20,76,77,78]); (**B**) InGaAs/InGaP core/shell nanowires array laser monolithically integrated on SOI: (a) Schematic of the nanowire array laser, (b) Electric field profile (|E|) of the fundamental cavity mode, (c) Tilted SEM images of the nanowire array laser and (d) individual NW (reprinted with permission of Ref. [19]); (**C**) InP nanowire array solar cells: (a) top-view and 30° tilt scanning electron micrographs of as-grown NWs, (b) SEM images of processed nanowires with a schematic of the individual devices, (c) optical microscope images of the solar cells, (d) solar cell I-V characteristics (reprinted with permission of Ref. [16]); (**D**) NWs array based nanomechanical biosensor for living cells induced forces detection: (a) scanning electron micrograph of the cells immobilized on the array, (b) fluorescence of the cell and reflection of the nanowire array, (c) distortion analysis as a function of time. (Reprinted with permission of Ref. [81]).

Figure 1 reports selected examples of nanowire array applications presented in literature, with cases of devices integrated in the individual nanowires composing the array. In particular, Figure 1A reports different examples of highly ordered NW arrays—both grown on selected areas and individually seeded—and it shows how the X-ray diffraction spectra and the visible photoluminescence emission of In_x_Ga_1−x_N arrays can be tuned by controlling the compositional range of the NW material, with *x* spanning from 0 to 1 [20,76,77,78]. Figure 1B reports InGaAs/InGaP core/shell NW array lasers monolithically integrated on SOI [19]. The integration of III-V semiconductor-based devices on silicon platforms by heteroepitaxy is particularly challenging due to the lattice constants and thermal expansion coefficients mismatch, leading to the formation of highly defective and reduced performing devices [79]. Bottom-up growth approaches have been demonstrated as valid techniques to growth high quality III-V NW materials on silicon [80]. Figure 1C reports an example of the application of NW assemblies for energy harvesting. The figure illustrates the realization of an InP NW array with each individual NW implementing a solar cell, reporting scanning electron micrographs and optical images of the device, as well as its electrical characterization [16]. Finally, an example of a nanomechanical biosensor based on an NW array is reported in Figure 1D [81].

However, we notice that the few examples reported so far do not provide an exhaustive picture of all successful NW array applications reported in the literature, especially taking into account the important issue of integration of III-Vs nanostructures with silicon platforms. For instance, InGaN-based NW LEDs fabricated on silicon substrate by self-assembly techniques with emission color control from blue to red have been reported [82]. Besides, to mention another few extremely promising applications, periodic gratings made of NW arrays coupled with planar Si photonic waveguides have been proposed [83], and NW arrays locally grown on silicon micro cantilevers for ultrasensitive gas sensors [84] were also reported.

**Figure 2:**
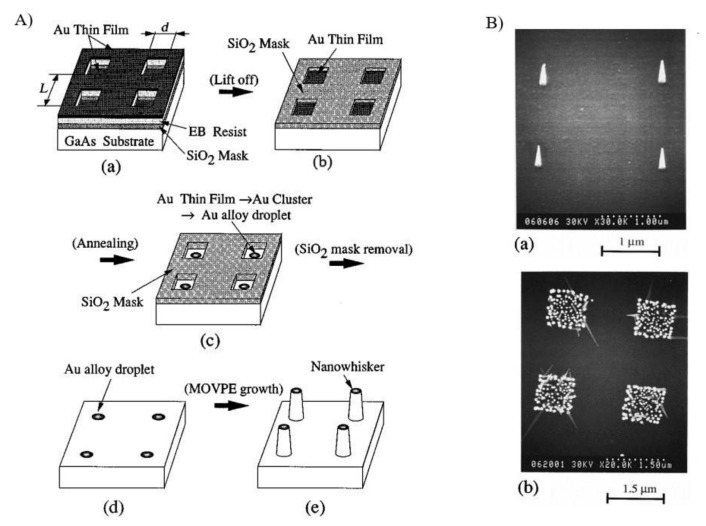
(**A**) Pictorial representation of the site-controlled growth method of nanowires reported in the first works on nanowire positioning (a) SiO_2_ mask formation by electron-beam lithography; (b) removal of Au atoms from the masked region by the lift-off process; (c) Au alloy droplet formation by annealing; (d) removal of SiO_2_ mask; (e) MOVPE growth of nanowhiskers; (**B**) scanning electron micrographs of the first nanowire positioning experiments, varying the dimension of the mask’s openings (a) individually seeded NWs, (b) growth on selected areas (reprinted with permission of Ref. [86]).

The first works on the controlled positioning of vertical nanowires started to appear during the 1990s, as a part of the pioneering works of Himura et al. on nanowire growth [85,86,87]. These papers were the first to report the use of substrate prepatterning to assist the growth of III-V semiconductor nanowires at defined locations in the substrate. In particular, by exploiting lithographically defined silicon oxide window masks, they realized patterns of gold nanoparticles acting as catalysts for nanowire growth in a vapour-solid-liquid process (this mechanism will be explained in detail later in the paper). Figure 2 shows a schematic representation of the site-control nanowires (called nanowiskers at that time) growth process reported in one of the first Hiruma’s works [86].

In the bottom-up nanowire growth approaches, substrate prepatterning may consist of realizing a pattern of metal nanoparticles on the substrate’s surface, in realizing an array of openings in an thin oxide mask deposited on the substrate’s surface, or in mixed approaches, as it was the case in Hiruma’s work [86]. As shown later, the substrate patterns provide a template to promote the controlled growth of nanowires at specific locations in the substrate and with specific sizes.

With rare exceptions [88], most of the processes for the fabrication of ordered arrays of vertical nanowires, especially when high quality and high pattern precision is required, include at least one stage of lithographic patterning as the fundamental step to define the pattern geometry. As a matter of fact, the final outcome—the nanowire array—is dramatically affected not only by the specific growth parameters ruling the kinetics and thermodynamics of the growth mechanisms, but also by the geometry and quality of the pattern employed to assist the nanowire growth [3,89,90]. This is the reason why, in the context of ordered nanowire array realization, the role of the substrate patterning deserves a dedicated and accurate investigation: reviewing the investigation efforts spent by the scientific community in this direction is actually the goal of the present work.

## 3. Bottom-Up Approaches to the Realization of Ordered Arrays of Vertically Aligned Semiconductor Nanowires

### 3.1. Nanowire Arrays Growth from the Bottom-Up

The bottom-up approaches for the realization of ordered arrays of semiconductor nanowires mainly rely on two prominent techniques: nanoparticle-assisted growth, which is at present the most commonly used technique for NW growth [5,45,91], and catalyst–free growth, often obtained by selective area epitaxy. Both these techniques enable nanowires growth at specific locations predefined by lithographic prepatterning of the substrate.

The discovery of the vapor–liquid–solid (VLS) process—namely, the metal nanoparticle-assisted vapour phase growth of nanowires—demonstrated for the first time in the 1960s by the work of Wagner and Ellis [39,92], was the first important milestone in nanowire growth research. Starting from the 1990s, following the inspiring results obtained in those years on carbon nanotubes [93,94], the nanowire VLS growth mechanism started to be studied extensively. This process was then proven to be a suitable technique to grow very high quality materials and, as mentioned earlier, the improvement in the control over the synthesis process allowed the realization of very high quality nanoscale devices in a single nanowire [41,42,44].

VLS growth exploits the eutectic reaction between metal catalyst nanoparticles and the semiconductor source materials in their vapor phase. Usually, gold is employed as a metal catalyst, as it does not oxidize in air and it supports the decomposition and gathering of the precursors for a broad range of materials systems [95,96]. Above the eutectic temperature for the target metal–semiconductor system and in the presence of the semiconductor precursors, a liquid metal–semiconductor eutectic alloy is formed, and the system continues to incorporate the semiconductor material until supersaturation. Upon the supersaturation of the liquid alloy, the semiconductor starts to nucleate and precipitate at the liquid-substrate interface beneath the catalyst, giving rise to the nanowire growth. The growth proceeds as long as the continuous transport of precursor components from the gas phase is ensured [1,91]. The process temperatures are relatively low (usually a few hundreds of degrees), as the eutectic temperatures of the metal–semiconductor alloys are usually lower than the metal melting temperature and the growth can also occur in the sub-eutectic point [97,98].

The VLS nanowire growth mechanism can be promoted starting from several deposition techniques, including metal−organic vapor phase epitaxy (MOVPE) [46,99,100], laser ablation [101], molecular beam epitaxy (MBE) [102,103], metal organic chemical vapor deposition (MOCVD) [5,6] and chemical beam epitaxy (CBE) [7,8,104]. As a matter of fact, the versatility of the VLS method makes it the actual dominant option for the growth of nanowires and heterostructuctured nanowires [1,5].

In VLS process, the size and the location of the nanowire are mainly determined by the size and location of the metal nanoparticle used as catalyst [105,106]. The use of commercial metal nanoparticles, available in different specific and well-controlled diameters, is the most diffuse method to achieve forests of randomly distributed nanowires uniform in size. This method offers an excellent nanowire size control with respect to other methods, such as laser ablation or thermal annealing of thin metal films. Noticeably, in order to achieve the precise control over the nanowire position and size in an engineered array, it is possible to assist the NW growth by employing prepatterned substrates, where the position and size of each individual metal catalyst nanoparticle (or selected growth areas) is defined by means of lithographic techniques. Of course, any approach to nanowire growth based on a metal catalyst may pose a drawback related to the introduction of metal contaminations during the growth process [107,108]. While this may represent a minor or even negligible issue for the demonstration of prototypical nanoelectronic devices based on individual nanowires [47,56], at a time it can represent a major obstacle for the integration of semiconductor nanowires with silicon technologies, because the use of gold introduces deep traps in silicon, thus deteriorating, for instance, the optoelectronic properties of the devices [109,110]. To bypass this potential obstacle, many efforts were devoted to the development of alternative growth schemes, avoiding the use of foreign metal seed particles as catalysts, and at least two classes of methods were proposed as alternatives to metal-catalyzed growth, namely, self-assisted growth and catalyst-free growth.

In self-assisted growth, VLS mechanisms are still employed, but the catalyst droplet is composed of one of the same elements composing the grown nanowire. Indeed, the self-assisted growth process is based on an accurate balancing between the precursor flows, which can be tuned in order to have an excess of one of the components, forming a liquid droplet which can act as a catalyst [1]. In the case of III-V semiconductor NWs, this sort of self-seeded growth exploits low melting temperature group III elements as a seed for the NW growth. For instance, indium is used to assist the growth of InAs [111] and InP [112], while gallium is used to assist the growth of GaAs [6]. Self-assisted growth has the advantage of high purity of the grown materials and relatively straightforward control of the NW diameter and position [1,89]. Catalyst-free methods are instead not mediated by any metal or intermediate phase, but rather employ crystal growth rate anisotropies to drive the preferential growth in one dimension [5]. This technique, often referred to as vapor-solid, has been demonstrated for the growth of Si nanowires [113], III-V semiconductor [109,114,115], and ZnO nanowires [116]. Catalyst-free growth can be performed, exploiting a self-assembled growth approach which typically relies on substrate-nanowire lattice mismatch, or exploiting a selective area approach, such as selective area growth (SAG) or selective area epitaxy (SAE). In the latter case, the growth is promoted only on localized areas of the substrate that are defined by employing a patterned dielectric mask onto the substrate [1], where randomly arranged pinholes [117] or lithographically defined openings [118,119] act as nucleation points for the NW growth.

Both metal-assisted and self-assisted or catalyst free nanowire growth have been successfully applied to the realization of highly ordered arrays of semiconductor nanowires. Notably, to this purpose, the different growth techniques are often employed using hybrid approaches where, for instance, selective area growth is applied to support catalyst-assisted growth [120,121,122]. In the context of the present review it is worth highlighting that, despite the specific nature of the growth method, the engineering of ordered nanowire arrays implies the use of pre-patterned substrates, which are employed as templates to control nanowire location and size. Depending on the specific growth technique, the pattern may consist of metal nanoparticles located on the bare substrate or deposited with the help of a growth mask (in the case of metal-assisted growth) or be made of an array of holes in a growth mask (in the case of selective-area and self-catalyzed growth).

### 3.2. Substrate Metal Patterning for Nanowire Array Growth

As previously mentioned, metal-assisted growth is one of the most commonly used techniques for semiconductor nanowire growth, and it is also the most employed method for the realization of ordered nanowire arrays [11,123]. The first pioneering works on site-controlled growth of nanowires, realized by Hiruma and coworkers in the 1990s [85,86,87], were indeed based on catalyst-assisted growth and exploited SiO_2_ window masks to define the location of the metal catalyst. Using this approach, squared patterns of NWs with a diameter of 100 nm and separated by 2 µm were realized for the first time.

The choice of the lithographic patterning technique for substrate preparation depends on the desired specifications of the array. The nanowires composing an engineered array can be individually seeded, or grown randomly on selected areas. In the latter case, for growth areas with a feature size above several hundreds of nanometers, or even micrometers, the catalyst pattern can simply be defined by resorting to micropatterning techniques (e.g., UV lithography or laser writing) and then the nanowire growth can be obtained by applying the same processes commonly employed for NW VLS growth on unpatterned substrates, e.g., thin metal layer deposition followed by thermal de-wetting or a standard deposition of metal colloidal nanoparticles [124]. However, studies on the role of the catalyst’s shape in an individually seeded nanowire suggest that for growth areas below a few hundreds of nanometers, a dedicated optimization of the growth parameters is definitively required [125] (See Section 3.1).

In fact, for individually seeded nanowires, nano-lithographic methods are used to define the position of each individual catalyst. Electron beam lithography and nano imprint lithography are the most commonly used techniques, and will be reviewed in the following sections.

#### 3.2.1. EBL Pre-Patterned Substrates for Nanowire Epitaxial Growth

The most common technique exploited for the definition of precisely located metal seeds for ordered nanowire array realization is electron beam lithography (EBL) [78,120,121,125,126].

This technique has the advantage of a very narrow resolution, reaching values below 10 nm [127], coupled with a very high flexibility regarding the pattern design [128,129]. Following this route, two different metal-patterning approaches can be implemented. The first one consists of directly patterning the metal catalysts on the growth substrate, by spin-coating the e-beam resist on the substrate, opening holes in the resist by EBL, and then proceeding to metal evaporation and liftoff [104,120,125,130].

The second approach consists of a mixed protocol, which combines metal-assisted and selected area growth and is often referred to as “selective-area vapour–liquid–solid growth” [120,121,122,131]. In this case, the first step is the deposition on top of the bare substrate of an amorphous thin film, a few tens of nm thick (usually SiO_2_, but also SiNx [131] and substrate’s native oxides [132] were used). The substrate covered with the amorphous film is then spin-coated with e-beam resist, electron beam patterned, developed and wet etched in buffered HF to produce position-defined openings in the mask, then metal thermal evaporation and liftoff follows [120,131]. In all these cases the metal catalyst particles have the shape of nanodisks, with a diameter and thickness optimized according to the case specific requirements, but usually in the range below 200 nm for diameter and below 20 nm for thickness [104,125].

**Figure 3:**
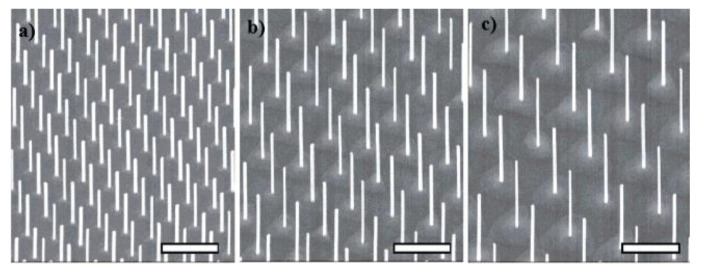
SEM tilted views of nanowire InAs NW arrays grown from EBLprepatterned substrates. The NWs have same diameters but different inter-wire distances: (**a**) 0.5 µm, (**b**) 0.75 µm and (**c**) 1 µm. The nanowires were grown from Au catalysts realized by electron beam lithography. Scale bar is 1 µm in each panel. (Reprinted with permission of Ref. [104]).

In Figure 3 some examples of InAs nanowire-ordered arrays grown on a prepatterned substrate realized by electron beam lithography are shown [104].

In realizing a vertical nanowire array, the EBL resolution can be considered in principle as a lower limit for the diameter of nanowires and their center-to-center distance. However, the resolution of the nanowire array after growth is affected by many other mechanisms taking place during growth, which require an accurate optimization. A further discussion regarding the pattern geometry role in nanowire array definition will be reported in Section 4.

#### 3.2.2. Nanoimprint Lithography Pre-Patterned Substrates

**Figure 4:**
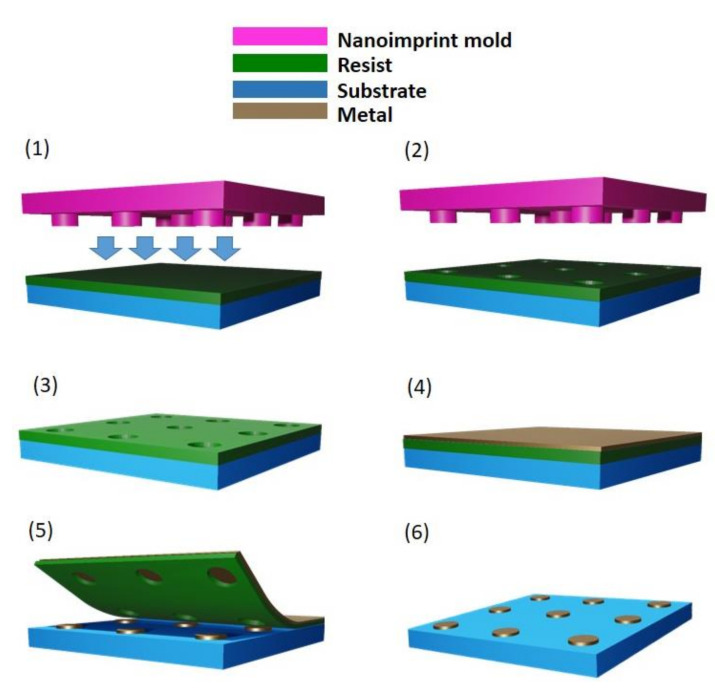
Schematics of substrate prepatterning process by means of nanoimprint lithography for metal-catalyzed nanowire growth: (**1**) a mold containing the pattern to be transferred is pressed against a layer of resist spin-coated on the substrate; (**2**) the mold is separated from the substrate, (**3**) the pattern is transferred to the resist mask; (**4**) a thin layer of metal is evaporated; (**5**) liftoff; (**6**) final metal catalyst pattern.

Nanoimprint lithography (NIL) is a nanopatterning method based on a compression molding approach. The process consists in pressing a rigid mold, with a nanometer-scale surface relief, into a thin layer of resist coated on top of a hard substrate. The resist layer is then hardened in order to allow the pattern to be transferred onto the material after the mold removal, making it suitable for other steps of fabrication, such as etching and deposition [133]. Compression molding has been used as a low cost fabrication technique in the upper micron region for many decades, but only since the end of the 90s nanoimprint has been demonstrated as a successful technique for nanofabrication, as patterning resolutions down to 25 nm were reached the first time [134]. Nowadays, nanoimprint allows to replicate features smaller than 10 nm over large areas with long-range order and it is considered as a promising approach to time and cost-effective fabrication of nanometer-scale patterns [135]. Nanoimprint lithography was first applied to define ordered arrays of metal-catalized vertically aligned semiconductor nanowire in 2004 [78] according to the process illustrated in Figure 4.

**Figure 5:**
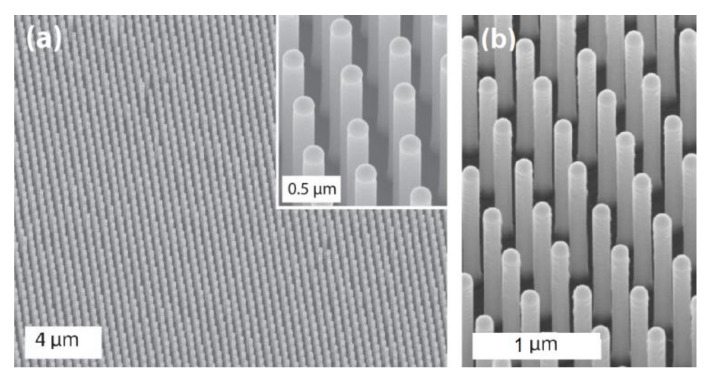
SEM tilted views of: (**a**) GaInP nanowire arrays with NW diameter D = 186, nm and pitch = 500 nm, grown by means of gold-catalyzed MOVPE on InP (111) substrates pre-patterned by using nanoimprint lithography (adapted with permission of Ref. [136], (**b**) InP NW array with period p = 400 nm, NWs diameter D = 138 ± 4 nm and length L = 1620 ± 40 nm; samples were grown on InP (111) B substrates previously pre-patterned by defining 160 nm diameter and 20 nm high Au catalyst particles in periodic arrays with a pitch of 400 nm using nanoimprint lithography. (Reprinted with permission of Ref. [137]).

Resorting to a statistical image analysis of the patterns realized using NIL and EBL, the authors demonstrated that NIL provided a control over diameter, location, and length of the nanowires with an accuracy very similar to that obtained using EBL. More recent works investigated strategies to improve the pattern preservation, reaching 100% of fidelity by using a metal assisted growth in combination with the use of a SiNx nanoimprint lithographic mask to define the catalyst positions [122]. Figure 5 shows the scanning electron micrographs of two examples of ordered III-V nanowire arrays realized by means of gold-catalyzed growth on InP substrates prepatterned by nanoimprint lithography [136,137].

Electron beam and nanoimprint lithography are the techniques which ensure the best precision for the realization of ordered arrays of semiconductor nanowires [138]. The major advantage of NIL over EBL, especially for larger scale applications, is the higher throughput, being NIL a parallel process; moreover, the stamp once produced (usually using EBL) can be reused repeatedly. This makes NIL a promising candidate for high-throughput and low-cost patterning, enabling full wafer processing for industrial applications. EBL remains the favorite choice for research and development applications as it provides the largest flexibility in defining the pattern.

### 3.3. Substrate Patterning Approach for Self-Assisted Growth and Catalyst-Free Selective Area Growth

Both self-assisted and catalyst-free growth by selective area epitaxy of ordered arrays of III-V nanowires rely on the use of dielectric mask templates to grow position-controlled nanostructures without the aid of metal droplets [109,139]. These techniques attract a lot of attention in view of the gold-free nature of the exploited growth method, which enables the compatibility of III-V nanowire growth processes with silicon technologies [85,89,140,141].

**Figure 6:**
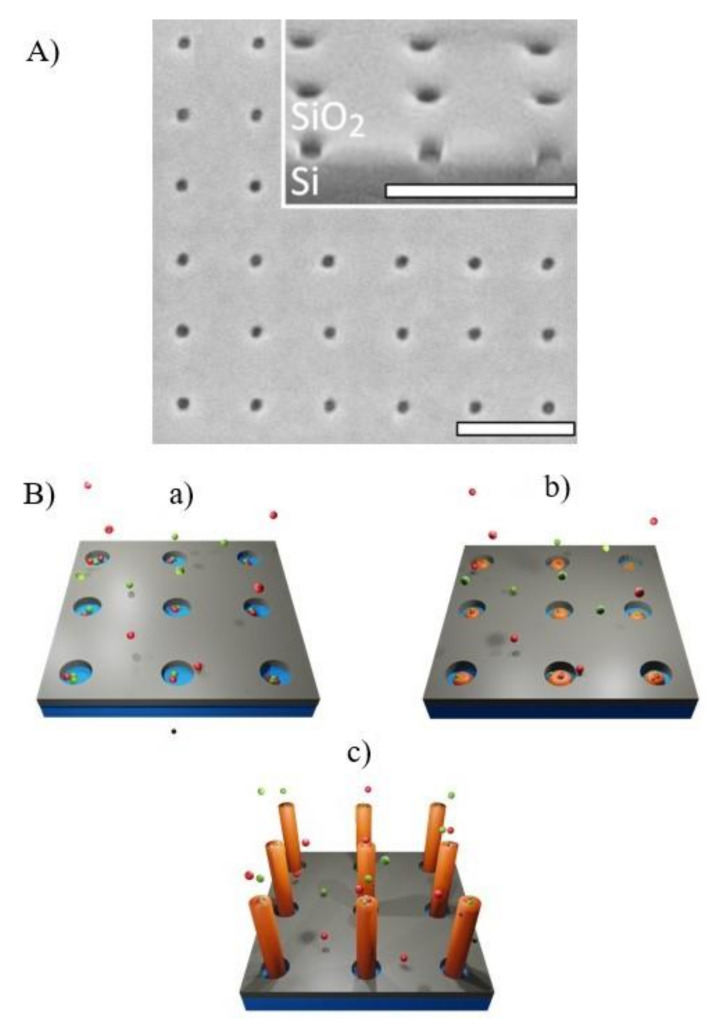
(**A**) Scanning electron micrograph of a nanoscale hole pattern realized on a 20 nm-thick thermally grown SiO_2_ layer, used as a mask template for nanowire selective area growth (Reprinted with permission of Ref. [149]). The pattern was realized by means of electron beam lithography followed by reactive-ion etching. The holes have a diameter of 20 nm and spacing of 125 nm and are arranged in a square lattice (the inset shows a tilted view of the pattern). The scale bar is 200 nm for all micrographs. (**B**) Pictorial representation of the selective area growth process: (a) the substrate, covered with the thin dielectric mask template, is exposed to the precursor gases; (b) the semiconductor material only grows selectively on the exposed regions of the substrate; (c) final selective area grown array.

The metal-free growth of III-V semiconductor NWs (both homogeneous and heterostructured nanowires) was indeed demonstrated on both III-V [142,143,144] and elemental semiconductors such as Si [145,146,147] and Ge [148]. While the full understanding of the peculiarities of each mechanism is still a matter of debate, the common aspect of these growth techniques—which is of interest for the scope of the present review—is that the mechanisms rely on the presence of nanoscale openings in an amorphous thin layer mask: these openings act as nucleation points for the growth. The scanning electron micrograph of a holes pattern in a SiO_2_ mask, acting as template in selective area growth of GaN nanowires, is shown in Figure 6a, while Figure 6b reports a pictorial representation of the process.

Similarly to the case of metal-catalyzed growth, also in metal-free approaches, the position, shape and dimensions of the openings in the amorphous oxide templates have a crucial role in determining the final pattern geometry [139,150]. Moreover, in this case, electron beam lithography is the most used technique to define the oxide template [120,151], although the use of nanoimprint has been proposed as a valuable alternative for large scale applications [152].

### 3.4. Alternative Nanopatterning Techniques

**Figure 7:**
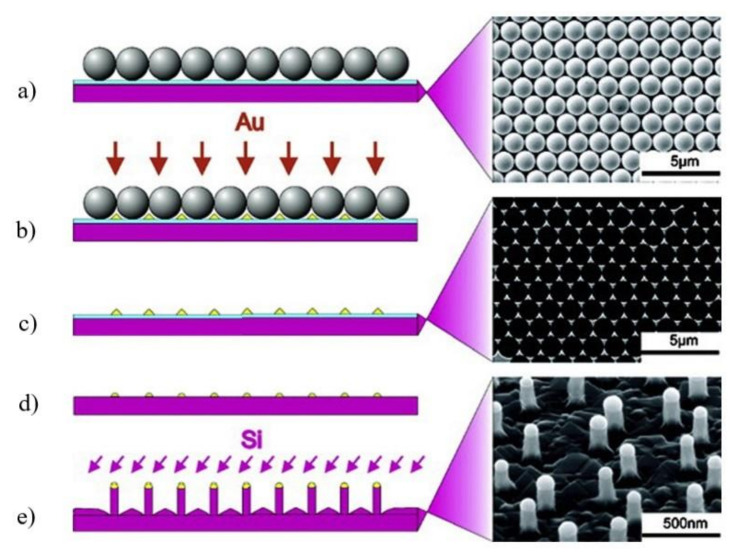
Nanosphere lithography applied to the preparation of ordered vertical Si NWs using gold-catalyzed VLS growth: (**a**) deposition of polystyrene nanoparticles on a silicon substrate covered with a 2 nm thick oxide layer (on the right a scanning electron image is reported); (**b**) thermal evaporation of gold; (**c**) removal of the nanospheres layer (on the right a scanning electron image of the patterned substrate is reported); (**d**) annealing and removal of the oxide layer; (**e**) NW growth (on the right a scanning electron image of the NW array is reported). Reprinted with permission of [155].

In the search for lower cost alternatives to electron beam and nanoimprint lithography, some other patterning techniques have been investigated for application in substrate pre-patterning for nanowire ordered array realization. Among them, nanosphere lithography is one of the most investigated [153] and it was applied to the growth of different semiconductor nanowires, including III-V materials [153,154,155]. Nanosphere lithography is a low cost patterning technique, which exploits the self-assembling, on the substrate’s surface of nanosized spheres, usually composed of polymeric materials, such as polystyrene, coated on the substrate as a suspension of monodispersed spherical colloids [156]. The nanospheres, under certain conditions and treatments, create a monolayer of closely packed nanospheres on the substrate surface, which can act as a mask for substrate pre-patterning. The produced patterns consist of hexagonally ordered arrays of elements with an approximate triangular shape and feature a size down to 100 nm [154,157]. Nanosphere lithography was applied in combination with both Au-catalyzed VLS growth [155] and catalyst-free selective area growth [153]. Figure 7 shows a pictorial representation of the nanosphere lithography substrate pre-patterning process, applied to the preparation of ordered vertical Si nanowires using Au-catalyzed VLS growth (left); on the right, scanning electron micrographs of some of the steps of the process are shown [155].

The results presented in the literature demonstrate that the technique imposes strict constraints on the pattern geometry and feature size and, moreover, that the produced patterns are affected by a variety of defects due to grain boundaries, edge defects, etc. [153,154,156] Nanosphere lithography can thus be considered as a noteworthy low-cost alternative to EBL and nanoimprint for substrate pre-patterning only in some specific cases, where the low flexibility in the pattern geometry is an acceptable compromise and in applications characterized by a certain tolerance toward defects. For example, this technique has been successfully applied for the realization of NW arrays for light management in photovoltaic applications [153]. However, in general, the fixed pattern geometry and the poor pattern fidelity make this technique unsuitable for the realization of fully engineered nanowire arrays and metasurfaces.

Another promising technique for substrate pre-patterning is block copolymer (BCP) lithography. The mechanism is similar to nanosphere lithography, but it exploits block copolymers self-assembly, instead of nanosphere self-assembly, for creating a thin mask on the substrate surface. Block copolymer self-assembling consists of a spontaneous organization of polymer molecules into well-defined regular features in the 5–100 nm range, driven by the minimization of the free energy [158]. This technique has been applied to the realizations of InAs nanowire arrays [159,160]. This technique is still immature for the realization of engineered nanowires arrays, but it is a very promising candidate for low-cost substrate patterning over large areas [123].

Another patterning technique, which has recently been applied in order to successfully nano-engineer a III-nitride nanowire array, is displacement Talbot lithography [161]. Displacement Talbot lithography is a new technique for patterning large areas with sub-micron periodic features, at lower costs with respect to EBL and nanoimprint. The technique exploits the Talbot effects, which consist of the creation of three-dimensional interference patterns by illuminating a periodic mask by coherent light [162]. This technique has been applied for dielectric mask nanofabrication for applications in the selective area growth of InGaN/GaN core-shell nanorods [161].

Some applications of interferometric lithography (IL lithography) for GaN nanowire growth by selective area growth on GaN films have also been reported. IL lithography allowed the realization of SiN masks, consisting of hexagonal patterns of apertures with a diameter around 220 nm and a pitch of 500 nm [163]. By using these techniques, the minimum nanowire diameter and array pitch achievable are limited by the IL lithography resolution. By resorting to shorter wavelength lasers and immersion lithography, it is expected to reach diameters in the 10–100 nm and pitches in the 100–200 nm [163,164]. Indeed, the fabrication of GaAs nanowire arrays, optimized for photovoltaic applications, having NW diameter of 300 nm and pitch of 600 nm, have already been reported by using deep UV lithography.

## 4. Influence of the Pattern Characteristics

The study of the role of the pattern characteristics, namely the metal seed size and the aspect ratio in metal-catalyzed growth, the hole sizes and aspect ratio in template assisted growth and the pitch, in enabling the reliable fabrication of uniform and controlled nanowire arrays, is sparsely addressed in the literature, but its crucial impact is fully recognized [3,89,125]. Some relevant information about the main mechanisms relating the pattern characteristics to the final array properties, as well as the connected experimental limitations in fabrication, can anyhow be gathered from the abundant literature in the growth field, which also includes some interesting recent papers and reviews [1,5]. Trying to put forward a comprehensive discussion including all the reported results, obtained for a large variety of nanowire materials, growth techniques and conditions, would be a very hard task, because the reported findings are most of the time dependent on the specific used growth conditions, adding a further complexity to the analysis.

In the following section we will focus our attention mainly on the results reported for arsenide and phosphide nanowires and we will try to provide some general insights into the role of the substrate prepatterning, identifying some generally valid trends which are independent on the specific growth conditions. If this allows us to shed some light on the occurring phenomena, at the same time it rules out the ambition of a quantitative study. We refer to other recent works for the description of the role of the other growth parameters, which also affect the final array properties [5].

### 4.1. Role of the Pattern’s Individual Element Shape

Both in catalyst-assisted and in catalyst-free growth, the individual element composing the pre-patterned substrate (a metal seed on the substrate or a hole in an amorphous oxide mask) has the shape of a nanodisk, with a diameter below a few hundred nanometers and a height below a few tens of nanometers. The choice of the size of the individual element composing the pattern affects the final NW array morphology.

#### 4.1.1. Role of the Pattern’s Individual Element in Defining the Nanowire Diameter

For the growth conditions at which only axial growth occurs, the nanowire diameter is defined by the geometric parameters of the individual element in the pre-patterned substrate. As previously mentioned the current substrate patterning techniques allow to define the catalyst seeds and etching mask openings in the scale of a few nm diameter. However, the patterning technology resolution can be assumed only as a rough estimation of the lower NW diameter and array pitch, as the individual pattern featured size often does not correspond precisely to the nanowire size [5]. In VLS nanowire growth of III-V semiconductor compounds and other semiconductor materials, the nanowire diameter depends in first approximation on the seed particle volume, which includes both the starting catalyst droplet volume and the amount of the source materials incorporated into the seed [8,78]. Detailed models to explain the mechanism have been proposed [165,166]. During the initial stages of the growth, the catalyst nanoparticles will melt and often expand as they become saturated with the source materials, causing the nanowire diameter to exceed the initial catalyst particle size of several nanometers [125,167]. The contact angle formed by the metal catalyst and the nanowire growth facet, which is correlated to the catalyst stability at the nanowire tip, also plays a crucial role [5], affecting the diameter [5,168] and in some cases also the crystal structure [169], and growth direction [170]. Similarly, in selective area growth, the nanowire diameters are defined by the dimension of the openings in the growth mask. Moreover, in this case, the smallest diameter obtained for self-catalyzed growth is around 10 nm, which was obtained with an accurate control of the contact angle. In general, reaching nanowire diameters in the sub-30 nm range by bottom-up growth—which would enable quantum confinement functionalities—is presently considered a challenging task, and the possibility to realize large area arrays of controlled nanowires in that scale range still needs optimization [5,171,172]. Moreover, changes in diameter along the NW body (tapering and radial vapor-solid growth) and local random or non-random fluctuations of the diameter are often observed.

#### 4.1.2. Role of the Pattern Individual Element Aspect Ratio

**Figure 8:**
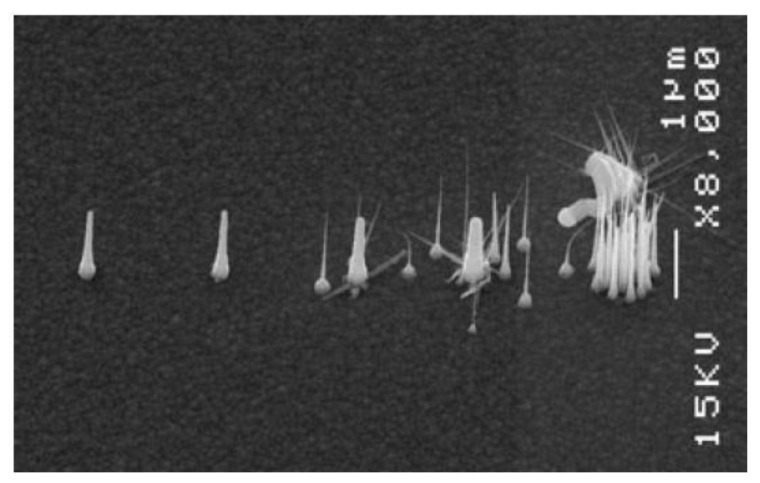
Scanning electron micrographs of InP nanowires grown from gold catalysts with different thickness-to-height ratios, showing the role of the catalyst’s aspect ratios in determining the properties of the grown NW array. The metal pattern realized on the substrate prior to growth consisted of five gold disks with a fixed thickness of 17 nm and varying diameters. Starting from the left, the catalyst diameters were: 50, 100, 200, 400, and 800 nm, corresponding to gold catalysts thickness-to-height ratios of about 1/3, 1/6, 1/12, 1/24, and 1/47. As shown in the image, for thickness-to-height ratios of 1/3 and 1/6, each individual catalyst originates a single nanowire. For lower aspect ratios, the catalysts are unstable and tend to split into many small catalyzing particles, giving rise to the growth of multiple inhomogeneous nanowires. (Reprinted with permission of Ref. [125]).

In metal-catalyzed growth, metal seeds in the form of very thin disks tend to break up, forming many small catalyst nanoparticles, acting as individual catalyst seeds for many nanowires, rather than supporting the growth of a single nanowire in a defined position. This effect is shown in Figure 8, which reports the micrograph of a set of InP nanowires grown from EBL-patterned gold catalysts with different diameters (50 nm to 800 nm) and a fixed height (17 nm). In particular, the thickness-to-diameter ratio seems to play the most important role: in the case of InP NW growth, ratios around 1/3 to 1/6 ensure a good catalyser stability, able to support the growth of a single nanowire [125,173]. Above that value, the catalyst splits in multiple nanoparticles and several inhomogeneous NWs originate from the same gold nanodisk.

The same phenomenon was observed for the Ni-catalyzed growth of vertical carbon nanofibers and nanotubes [174,175], as well as for ZnO NWs [176].

A similar effect was recently confirmed in the case of self-catalyzed growth of ordered III-V nanowire arrays grown on silicon, while using a lithographically defined pattern of holes in a silicon oxide mask as a template for catalyst droplet positioning [3,145]. Moreover, in that case, the aspect ratio (diameter-to-height ratio) rather than the hole diameter or height alone was identified as the key parameter. Moreover, the reported range of optimal values, i.e., the values which guarantee the best yield, are very similar to the case of metal catalyst growth. In fact, patterns made of holes with a diameter/height ratio between 4 and 6 resulted as the optimal ones [3,145,177]. The mechanism was confirmed by investigating thicknesses between 10 and 20 nm, diameters from 30 to 90 nm, and several hole-to-hole distances ranging from 200 nm to 2 µm.

**Figure 9:**
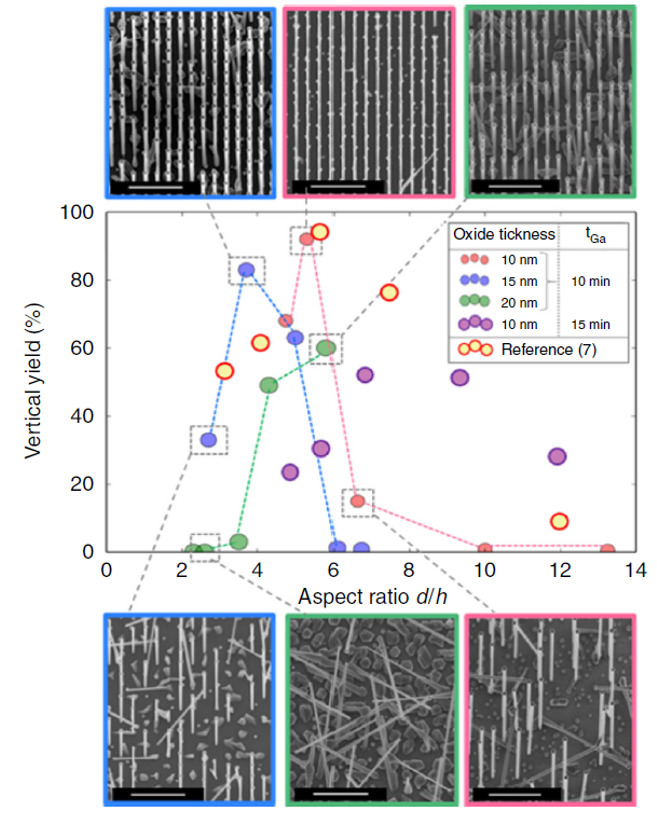
Results of an optimization study of the yield of vertical GaAs nanowires, grown by a self-catalyst method on silicon substrate by the hole aspect ratio in the SiO_2_ mask template used to assist the growth. Different values of the aspect ratio d/h were obtained using three different oxide thicknesses h of 10, 15, and 20 nm (corresponding to the red, blue, and green data points, respectively), and different diameters d. All samples were grown under the same growth conditions. The central graph shows the dependence of the vertical yield for the nanowire arrays as a function of the hole aspect ratio, while the inserts show 20° titled SEM micrographs of the samples with the best (upper part images) and worst (lower part images) yields. The maximum yields were obtained for the aspect ratios between 4 and 6 for all three oxide thicknesses used. Outside this range, the yield is compromised by the presence of parasitic growth. The central graph reports also the results of Ref. [104], following the same trend and represented by the yellow points (Reprinted with permission of Ref. [3]).

Figure 9 reports the results of an optimization study of the yield of vertical GaAs nanowires, defined as the ratio of the total NW number to the total pore number (or the fraction of holes filled with a vertical NW), grown by a self-catalyst method on silicon substrate, as a function of the hole aspect ratio [3].

In the authors opinion, the symmetrical filling of the hole by the catalyst droplet accounts for the optimal nanowire growth behaviour in the specified aspect ratio range [3]. Another study [178] exploring the effect of the dielectric mask’s openings shape on the yield of self seeded InAs nanowire arrays grown on silicon, investigated hole aspect ratios in the range below about 5. A 45–55 nm thick SiO_2_ mask was pre-patterned with the holes of a diameter ranging from 80 nm to 220. Similar to the previously reported study, and also in this case, yields above 80% were obtained for hole aspect ratios ranging in the 5–3.5 interval. For lower hole aspect ratios (lower opening diameters), a drastic decrease in the yield was observed, which was interpreted by the authors as a reduction in nucleation probability. In the explored range, the opening diameter, instead, did not affect the diameter or length of the grown nanowires, which were mainly determined by the specific growth parameters.

This mechanism is less investigated in the case of catalyst-free growth. A paper reporting about InAs NWs grown on SiO_2_/Si prepatterned substrates by means of catalyst-free growth [179] indicates high pattern yields (about 90%) for variations in the hole diameters in the range 40–100 nm (for the same hole depth of 18 nm), i.e., aspect ratios between 2.2 and 5.5, independently from the pattern pitch in the 250–5000 nm range. Moreover, in this case, the authors did not observe a remarkable diameter dependence on the growth mask hole diameters, and variation in this parameters from 40 to 100 nm resulted in a variation in the nanowire diameters from 133 to 143 nm.

#### 4.1.3. Role of the Pattern Individual Element Shape in Improving the Pattern Fidelity 

**Figure 10:**
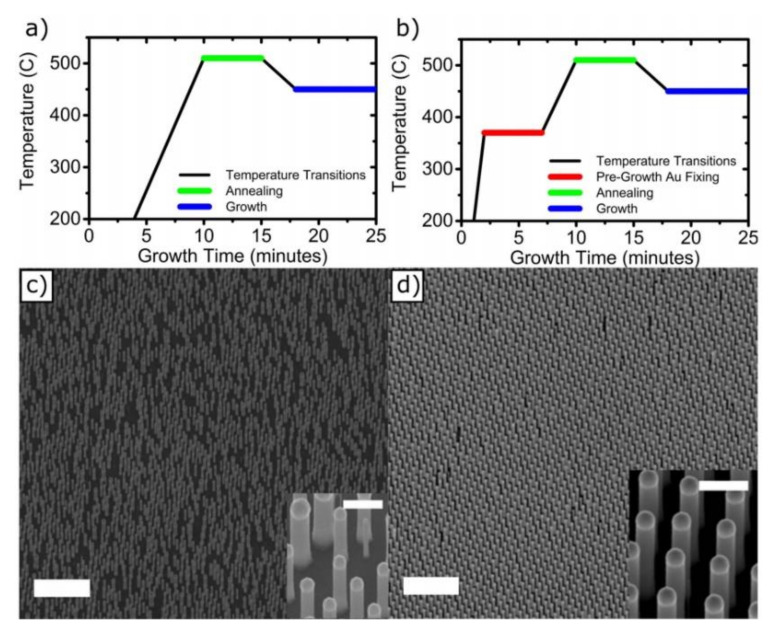
Effect of pre-annealing step in improving the vertical yield and uniformity of ordered InP NW arrays grown by the gold-catalyzed VLS method. The figure reports the process temperature evolutions and scanning electron microscope images of grown samples without including ((**a**)–(**c**)) and including ((**b**)–(**d**)) a pre-annealing step of the lithographically pre-patterned substrate. The samples were prepared for NW growth by defining 160 nm diameter and 20 nm high Au catalyst particles in periodic arrays with a period of 400 nm using nanoimprint lithography on InP (111)B substrates. Without the pre-annealing step (**a**) the au droplets can split, move and merge with other nanoparticles, causing missing nanowires in the pattern and deviations from the defined positions (**c**). The addition of a pre-annealing thermal treatment, at intermediate temperatures with respect to the growth temperature (**b**), enhances the adhesion of Au catalysts to their position, preventing them from moving across the substrate surface and improving the pattern fidelity (**d**) (Reprinted with permission of Ref. [173]).

One of the main mechanisms which severely affects the pattern fidelity in catalyst-assisted nanowire growth, especially when the nanowires are relatively thick (about 200 nm in diameter) [170], consists of the process of migration, splitting and merging of the Au seeds during the growth, which occurs as a consequence of the temperature values associated with the growth process. This causes the growth of nanowires at different locations with respect to the initial catalysts pattern, and also induces remarkable diameter inhomogenities in the final array [122,125,173]. Otnes et al. demonstrated that pre-anneal nucleation step in the growth chamber, before the standard NW growth scheme, is a useful strategy to reduce these effects and increase the pattern preservation [122]. This step of the substrate consists of a thermal treatment, at a temperature below the intended growth temperatures, performed in a controlled group III precursor flow. This step should reduce the melting temperature of the Au alloy droplet and promote a reaction between the Au catalysts and the substrate below them. This results in the formation of depressions in the substrates, preventing the droplet diffusion on the substrate surface and improving the pattern yield [122,173]. Figure 10 reports the comparison of two catalyst-grown InP nanowire arrays, without a pre-annealing step (left) and with a pre-annealing step (right).

An alternative method to obtain a very high yield in catalyst-assisted growth is to adopt a combined approach, which exploits selective area growth, combined with a catalyst-assisted method [77].

Notably, the optimal control of the individual element geometry of the pattern is not sufficient to guarantee a very high yield in the final NW array: several other growth parameters need to be optimized, as will be briefly discussed in the sections that follow.

### 4.2. Role of the Pattern Pitch on the Axial and Radial Growth Rate

One of the most investigated phenomena in the III-V nanowire array growth—both experimentally and theoretically—is the dependence of the array morphology on the pattern pitch, i.e., the center-to-center distance between neighboring seeds. This phenomena is not exclusively important for the realization of nanowire-ordered arrays or metasurfaces, but it also affects in general the growth of random nanowires, especially in the case of very dense assemblies. It has been demonstrated that the pattern pitch strongly influences the nanowire morphology as it can alter the local availability of the precursor species and thus induces variations in the growth kinetics [89,146,180].

One of the most investigated effects is the dependence on the pattern pitch of the axial and radial growth rate [89,104,179,181,182,183]. It is in fact observed that both the axial and radial growth rates decrease with the decreasing pitch, especially in dense arrays. This phenomenon, reported for different growth techniques (MBE and CBE) and for both metal-assisted [181,182] and catalyst-free approaches [169], has been ascribed to two main concomitant effects: shadowing of the direct group-III precursor flux impinging on the nanowire sidewalls [131,181,182,184], and the shared substrate diffusion areas of adatoms between different NWs, leading to a competition in the absorption process between different nanowires, thus lowering the growth rate [89,104,179,180,182,183]. This effect can also cause nanowire size inhomogeneities in dense arrays, as the growth rates of NWs in the pattern edges is higher with respect to the central regions [184]. In the case of InAs grown by gold-catalyzed molecular beam epitaxy on InAs(111)B substrates, the minimum reported pitch to consider the growth of each nanowire as independent from neighbors is 2 µm [182]. Moreover, in the case of InAs NWs grown on SO_2_/Si prepatterned substrates by means of catalyst-free growth, the minimum pitch value which does not affect the NW diameter and growth rate was around 1.5–2 µm [179]. Below this distance, accurate optimization of the growth parameters is required. In the work [130] a synergetic growth regime has been observed in Au-assisted nanowire growth, leading to an increase in the growth rate for the decreasing wire-to-wire distance, but this effect has not been demonstrated to be generally active [89,183].

### 4.3. Impact of Tapering

It is worth mentioning another important morphological feature of NWs that can have a significant impact when it comes to engineering a NW array, though not directly related to the pre-patterned substrate characteristics: the NW tapering. Tapering consists of a monotonic variation of the nanowire diameter along the axis, intentional or not intentional, which results in NWs with inclined sidewalls. Both positively and negatively (inversely) tapered NWs have been reported [5,185,186]. The control of this property is very important in the context of NW metasurface realization, as several studies suggest the crucial role of tapering in modulating and enhancing the ability of NW arrays to manipulate light beam propagation [18,34,187]. In the case of catalyst-assisted growth, the tapering control can be obtained by controlling the value of the droplet contact angle during growth, by varying the precursor gas partial pressure in the growth chamber and by controlling the droplet volume [169,185,186,188,189,190]. Temperature variations during the growth can cause variations in the catalyst volume, as they affect the semiconductor component incorporation in the catalyst’s droplet [5,191]. Tapering is also observed when the growth rate on the wire sidewalls is not negligible with respect to the axial growth. In other words, tapering can be regarded as a parameter to evaluate the relationship between the radial growth rate and the axial one.

## 5. Conclusions

Bottom-up grown semiconductor nanowires are very well-established building blocks in many field of research, but to unleash the full potential of their technological applications it is necessary to master the fabrication processes of assemblies made of a large number of nanowire, in order to reach a precise control of the position, size, and shape of the individual nano objects on the substrate. In this review, we have reported the state of the art of substrate pre-patterning for the realization of ordered arrays of vertically aligned semiconductor nanowires by means of bottom-up growth approaches. Focusing on III-V semiconductor nanowire systems, and in particular III-arsenides (III-As) and III-phosphides (III-P) semiconductors, we sistematically discussed the role of the substrate pattern characteristics in determining the nanowire array properties: we analyzed the specific role of all pattern morphological characteristics in the final array properties and yields, and we discussed the opportunities and limitations of the currently available nanofabrication techniques. In the review it is clarified in detail how the size and shape, and specifically the aspect ratio, of the individual metal seeds in metal-catalyzed growth and of the thin oxide template openings in selective area growth play the most important role, together with the pattern pitch.
